# Draft genome and sequence variant data of the oomycete *Pythium insidiosum* strain Pi45 from the phylogenetically-distinct Clade-III

**DOI:** 10.1016/j.dib.2017.10.047

**Published:** 2017-10-26

**Authors:** Weerayuth Kittichotirat, Preecha Patumcharoenpol, Thidarat Rujirawat, Tassanee Lohnoo, Wanta Yingyong, Theerapong Krajaejun

**Affiliations:** a Systems Biology and Bioinformatics Research Group, Pilot Plant Development and Training Institute, King Mongkut's University of Technology Thonburi, Bangkhuntien, Bangkok, Thailand; b Department of Biomedical Informatics, University of Arkansas for Medical Sciences, Little Rock, AR, USA; c Research Center, Faculty of Medicine, Ramathibodi Hospital, Mahidol University, Bangkok, Thailand; d Department of Pathology, Faculty of Medicine, Ramathibodi Hospital, Mahidol University, Bangkok, Thailand

**Keywords:** *Pythium insidiosum*, Pythiosis, Draft genome, Sequence variant

## Abstract

*Pythium insidiosum* is a unique oomycete microorganism, capable of infecting humans and animals. The organism can be phylogenetically categorized into three distinct clades: Clade-I (strains from the Americas); Clade-II (strains from Asia and Australia), and Clade–III (strains from Thailand and the United States). Two draft genomes of the *P. insidiosum* Clade-I strain CDC-B5653 and Clade-II strain Pi-S are available in the public domain. The genome of *P. insidiosum* from the distinct Clade-III, which is distantly-related to the other two clades, is lacking. Here, we report the draft genome sequence of the *P. insidiosum* strain Pi45 (also known as MCC13; isolated from a Thai patient with pythiosis; accession numbers BCFM01000001-BCFM01017277) as a representative strain of the phylogenetically-distinct Clade-III. We also report a genome-scale data set of sequence variants (i.e., SNPs and INDELs) found in *P. insidiosum* (accessible online at the Mendeley database: http://dx.doi.org/10.17632/r75799jy6c.1).

**Specifications Table**

**Table:** 

Subject area	Biology
More specific subject area	Microbiology, Genomics
Type of data	Genome sequence, Sequence variants, Phylogenetic relationship
How data was acquired	IlluminaHiSeq 2500 Next Generation Sequencing Platform
Data format	Assembled genome sequence, Sequence variants [i.e., single-nucleotide polymorphisms (SNPs) and small insertions and deletions (INDELs)], Phylogenetic tree
Experimental factors	Genomic DNA was extracted from the *Pythium insidiosum* strain Pi45, which is categorized in the phylogenetically-distinct clade-III.
Experimental features	A rDNA-based phylogenetic tree of *P. insidiosum* was generated. Genome of the *P. insidiosum* strain Pi45 was sequenced and assembled. The reference genome sequence of the *P. insidiosum* strain Pi-S was mapped with sequence reads from the *P. insidiosum* strain Pi45 to identify SNPs and INDELs.
Data source location	The organism was isolated from a patient with pythiosis in Thailand.
Data accessibility	The draft genome sequence of the *P. insidiosum* strain Pi45 (also known as MCC13) has been deposited in the Data Bank of Japan (DDBJ) under the accession numbers: BCFM01000001-BCFM01017277. The sequence variant data (i.e., SNPs and INDELs) of the *P. insidiosum* strain Pi45 is accessible online at the Mendeley database (http://dx.doi.org/10.17632/r75799jy6c.1).

**Value of the data**

• The first draft genome sequence of a *P. insidiosum* strain from the rDNA-based phylogenetic-distinct clade-III is now available.
• Draft genome data of the *P. insidiosum* strain Pi45 will be valuable for comparative genomic studies of *Pythium* species and related oomycetes.
• Sequence variant data (i.e., SNPs and INDELs) will be applicable for identification of the organism, genetic polymorphism analyses, genotype-phenotype association studies, and epidemiological exploration.

## 1 Data

**Fig. 1:**
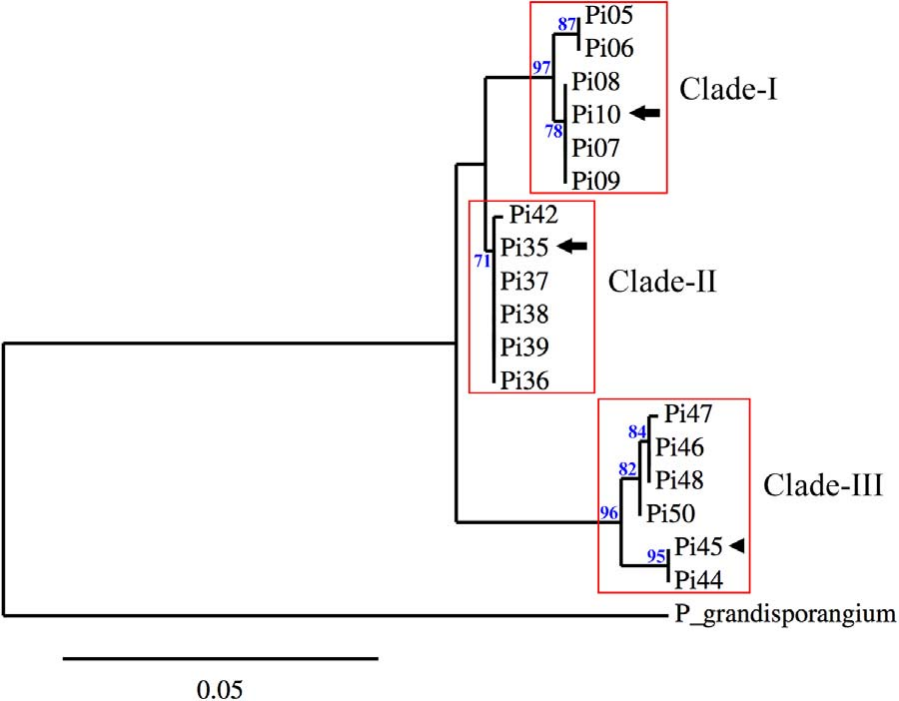
Phylogenetic relationship of *Pythium insidiosum*: the rDNA-based maximum-likelihood phylogenetic tree categorizes 18 strains of *Pythium insidiosum* into three distinct clades: Clade-I, Clade-II, and Clade-III. Description of each strain of *P. insidiosum* can be found in Table 1. The arrows indicate the strains [i.e., CDC-B5653 (labeled as Pi10) and Pi-S (labeled as Pi35)] where genome sequences are publically available, while the arrow head indicates the strain Pi45 where genome data is reported here. The rDNA sequence from *Pythium granisporangium* (accession number: AY151182) is included as an outgroup. Branch support values of greater than 70% are demonstrated at the nodes. Nucleotide substitution per site is shown at the bottom.

*Pythium insidiosum* is a member of the oomycetes, a unique group of fungus-like microorganisms belonging to the Kingdom Stramenopiles [1]. *P. insidiosum* is distinguished from other oomycetes by its capacity to infect humans and animals [1], [2], [3]. The infectious condition called ‘pythiosis’ caused by this organism usually leads to life-long disability or death in affected individuals [2], [3], [4], [5]. Genome sequence is a powerful resource that can be used to explore an organism of interest at the molecular level. Two draft genomes of the *P. insidiosum* strains CDC-B5653 [6] and Pi-S [7] are available in the public domain. *P. insidiosum* can be divided into three distinct clades: Clade-I (strains from Americas); Clade-II (strains from Asia and Australia); and Clade–III (strains from Thailand and the United States) (Table 1; Fig. 1). The strain CDC-B5653 (labeled as Pi10) is placed in the Clade-I, whereas the strain Pi-S (labeled as Pi35) is placed in the Clade-II (Fig. 1). The genome of *P. insidiosum* from the distinct Clade-III, which is distantly-related to the other two clades, is lacking. Here, we report genome data of the *P. insidiosum* strain Pi45, isolated from a Thai patient and categorized as Clade-III (Fig. 1). We also report a genome-scale data set of sequence variants (i.e., SNPs and INDELs) found in *P. insidiosum*.

**Table 1:** Eighteen strains of *Pythium insidiosum* used for generation of the rDNA-based phylogenetic tree. Strain identification numbers, reference numbers, host sources, geographic origins, assigned phylogenetically-distinct clades, and accession numbers of the rDNA sequences of all strains are summarized in the table. ‘*’ indicates the strains, including Pi45, where genome sequences are publically available.

**ID**	**Reference ID**	**Source**	**Country of origin**	**Clade**	**Accession number**
Pi05	CBS 575.85	Equine	Costa Rica	I	AB971178
Pi06	CBS 574.85	Equine	Costa Rica	I	AB971179
Pi07	CBS 573.85	Equine	Costa Rica	I	AB971180
Pi08	CBS 580.85	Equine	Costa Rica	I	AB898107
Pi09	CBS 101555	Equine	Brazil	I	AB971181
Pi10*	ATCC 200269	Human	USA	I	AB898108
Pi35*	Pi-S	Human	Thailand	II	AB898124
Pi36	ATCC 64221	Equine	Australia	II	LC199883
Pi37	ATCC 28251	Equine	Papua New Guinea	II	LC199884
Pi38	CBS 101039	Human	India	II	AB898125
Pi39	CBS 702.83	Equine	Japan	II	LC199885
Pi42	CR02	Environment	Thailand	II	AB971184
Pi44	MCC 17	Human	Thailand	III	AB971185
Pi45*	MCC 13	Human	Thailand	III	AB971186
Pi46	SIMI 3306-44	Human	Thailand	III	AB971187
Pi47	SIMI 2921-45	Human	Thailand	III	AB971188
Pi48	SIMI 4763	Human	Thailand	III	AB971189
Pi50	ATCC 90586	Human	USA	III	AB971190

## 2 Experimental design, materials and methods

### 2.1 rDNA-based phylogenetic tree

rDNA sequences from the strain Pi45 and 17 other strains of *P. insidiosum* were retrieved from the NCBI database (Table 1). The rDNA sequence from *Pythium grandisporangium* (accession number: AY151182) served as an outgroup. All rDNA sequences were subjected to phylogenetic analysis, using an array of online tools at www.phylogeny.fr [8], [9], [10], [11], [12], [13].

### 2.2 Genome sequencing and assembly

Genomic DNA of the *P. insidiosum* strain Pi45 was extracted [14] and processed to prepare one paired-end library for genome sequencing, using the IlluminaHiSeq 2500 platform (Yourgene Bioscience, Taiwan). Raw reads underwent quality trimming (minimal read length, 35 bases) by CLC Genomics Workbench (http://www.clcbio.com/). Adaptor sequences were removed by Cutadapt 1.8.1 [15]. A total of 33,692,522 adaptor-removed, quality-validated reads, equivalent to 3,488,072,978 total bases, were subjected to genome assembly by Velvet 1.2.10 [16]. The assembled genome consisted of 65,230,783 bases (‘N’ composition, 0.6%) in 17,277 contigs (average length, 3776 bases; range, 300–209,930 bases; *N*50, 14,374 bases). Assessment of the resulting draft genome sequence by CEGMA [17], [18] showed 78.6% genome completeness. A total of 26,058 open reading frames were predicted by MAKER2 [19].

### 2.3 Identification of sequence variants

A total of 7,843,910 adaptor-removed quality-validated reads (23.3% of all reads), derived from the *P. insidiosum* strain Pi45, can be aligned to the reference genome of the *P. insidiosum* strain Pi-S [7], using the Burrows-Wheeler Alignment tool [20]. A total of 865,332 variants (i.e., SNPs and INDELs) were identified by FreeBayes [21].

### 2.4 Data accessibility

The draft genome sequence of the *P. insidiosum* strain Pi45 (also known as MCC13) has been deposited in the Data Bank of Japan (DDBJ) under the accession numbers: BCFM01000001-BCFM01017277. The sequence variant data (i.e., SNPs and INDELs) of the *P. insidiosum* strain Pi45 can be accessible online at the Mendeley database (http://dx.doi.org/10.17632/r75799jy6c.1).
